# A novel network based linear model for prioritization of synergistic drug combinations

**DOI:** 10.1371/journal.pone.0266382

**Published:** 2022-04-05

**Authors:** Jiaqi Li, Hongyan Xu, Richard A. McIndoe

**Affiliations:** 1 Center for Biotechnology & Genomic Medicine, Augusta University, Augusta, Georgia, United States of America; 2 Department of Population Health Sciences: Biostatistics & Data Science, Medical College of Georgia, Augusta University, Augusta, Georgia, United States of America; University of Pittsburgh, UNITED STATES

## Abstract

Drug combination therapies can improve drug efficacy, reduce drug dosage, and overcome drug resistance in cancer treatments. Current research strategies to determine which drug combinations have a synergistic effect rely mainly on clinical or empirical experience and screening predefined pools of drugs. Given the number of possible drug combinations, the speed, and scope to find new drug combinations are very limited using these methods. Due to the exponential growth in the number of drug combinations, it is difficult to test all possible combinations in the lab. There are several large-scale public genomic and phenotypic resources that provide data from single drug-treated cells as well as data from small molecule treated cells. These databases provide a wealth of information regarding cellular responses to drugs and offer an opportunity to overcome the limitations of the current methods. Developing a new advanced data processing and analysis strategy is imperative and a computational prediction algorithm is highly desirable. In this paper, we developed a computational algorithm for the enrichment of synergistic drug combinations using gene regulatory network knowledge and an operational module unit (OMU) system which we generate from single drug genomic and phenotypic data. As a proof of principle, we applied the pipeline to a group of anticancer drugs and demonstrate how the algorithm could help researchers efficiently find possible synergistic drug combinations using single drug data to evaluate all possible drug pairs.

## Introduction

Drug combination therapy is becoming an important tool in cancer treatment. With the emergence of cancer cell resistance to the front line of anticancer drugs and the slow process of new drug discoveries via traditional drug developing methods, synergistic drug combinations offer a potential treatment optimization against drug resistance and side effects. As a result, there is an increasing interest in screening the expansive combination of both approved drugs and other potential therapeutic agents. A synergistic drug combination means the overall therapeutic effect of the combination is greater than the simple sum of effects caused by individual drugs [1].

To find synergistic drugs, both *in vitro* and *in silico* methods have been widely used to screen potential drug combinations. Most *in vitro* methods are based on previous knowledge of the drugs, such as the drug target, drug side effects, and drug chemical structure. This is the most common method used by many academic laboratories in their drug research. Additionally, high-throughput screening of numerous drug combinations to profile their phenotypic effects on cell lines or primary patient-derived cells has also been used [2,3]. Many pharmaceutical companies and translational researchers have invested significant resources to screen thousands of drug combinations. Alternatively, a number of computational approaches have been developed to help prioritize drug pairs from many candidates in order to minimize cost and the time of high-throughput screening methods as well as increase the efficiency of the experimental design. Lezon’s lab uses Probabilistic Matrix Factorization (PMF) to predict the effects of drug combinations in a full symmetric matrix, in which each row and each column of the matrix corresponding to a drug, and the elements are the effects of the drug combination. They successfully predicted the missing values of the rest of the drug pairs by knowing the effects of only 50% of drug combinations [4]. DECREASE predicts the full dose-response matrices by measuring only the matrix diagonal of drug combinations [5]. They decrease cost by either decreasing the number of the tested drug combinations or minimizing the size of the dose-response matrices.

However, there are two significant issues with these methods that prevent drug combination research from effectively exploring new products for clinical operation. First, they all rely heavily on previously known knowledge of the drug. In reality, the effects of most of the drugs are not fully understood with some of them misguided due to the limitations of the scientific methods used by the researcher [6,7]. Additionally, due to the limited number of known drugs, the investigators have been restricted to a small pool of candidates. At the same time, the current standard for evaluating drug combination synergistic effects requires a multi-dose-response matrix experiment. The low-throughput nature of this experiment makes screening a large number of drugs costly and slow. Second, the number of conceivable drug combinations increases rapidly along with the number of drugs in consideration, even though the high-throughput screening methods combined with *in silico* computational algorithms substantially improved the capacity of the drug combination screening. Considering the number of available drugs and small molecules, dose ranges, and cell lines for screening, the possible combinations increase exponentially. Thus, it requires extensive resources and instrumentation and easily exceeds the number of resources in an academic laboratory.

For these reasons, several computational approaches that prioritize the synergistic drug combinations based on individual drug effects have been published. A community based-competition called the DREAM Challenge was launched to help the development of drug combination prediction methods [8,9]. Several methodologies show that their predictive ability is significantly better than random. Additionally, they find that the gene expression profile from individual drug treatment can be used to predict the effects of drug combinations. At the same time, the transcriptional and phenotypic responses have been reported to correlate with each other [10]. Furthermore, Diaz et al observed that the correlation of gene expression of monotherapies is associated with the synergy of the combinations in their dataset [11]. So it is reasonable to expect that gene expression profiles can provide the predictive power for drug combination prediction.

Recently, pharmaceutical companies created a large number of small molecules for screening. Several consortia have generated large-scale data repositories, such as gene expression data, to store molecular information about the response of many human cell lines to perturbations from both FDA-approved drugs and small molecules (e.g., LINCS and CTD^2^) [12,13]. The Library of Integrated Network-based Cellular Signatures (LINCS) project used the L1000 technology [14], screened more than twenty seven thousand perturbations (e.g. small molecules and drugs), and produced 1.3 million gene expression profiles using a panel of cell lines [12]. The data generated for this project includes gene expression data before and after treating multiple human cell lines with these drugs/molecules. Given the scope and size of this database, integrating data from a single drug treatment into an algorithm to prioritize possible synergistic drug combinations and provide the molecular signatures of many known drugs and uncharacterized compounds will offer a cost and time-saving strategy to find synergistic drug combinations in these perturbations. The primary aim of this study is to develop an unbiased, high-performance enrichment model for drug combinations that uses single drug-treated data.

**Fig 1:**
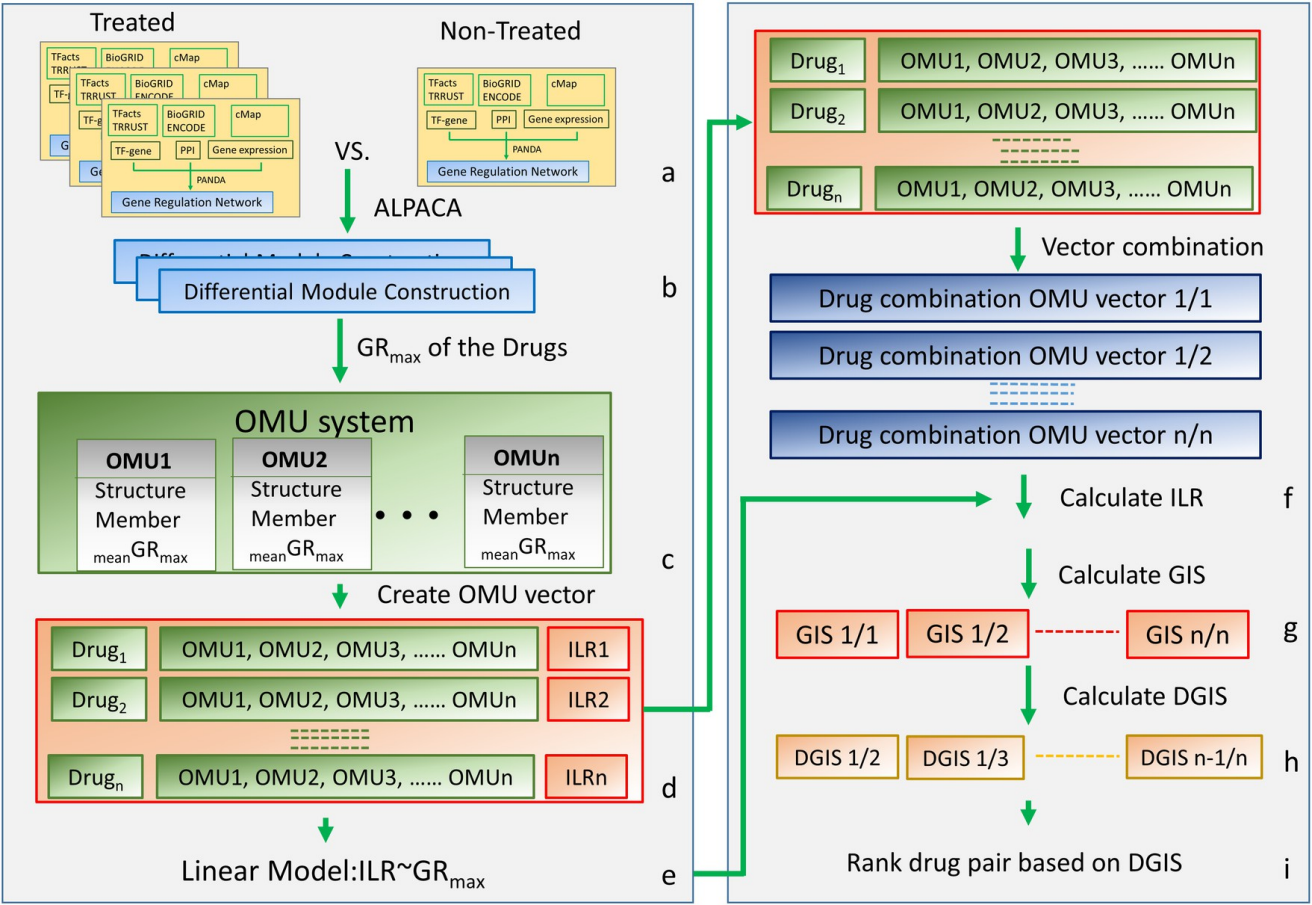
Schematic of the algorithm pipeline. (a) Re-construction of GRNs. (b) Generation of differential network. (c) Create an OMU system. (d) Create the drug OMU vector. (e) Linear model (f) Calculate the ILR for combined OMU vector. (g) GIS calculation (h) DGIS calculation (i) Sorting based on DGIS.

To achieve this goal, we developed an Operational Module Unit (OMU) system and then use this system to build a linear model to prioritize drug pairs with synergistic effects from all possible single drug combinations (Fig 1). We use the OMU system to connect the cell gene network alterations with the phenotype changes. The OMU system will allow us to directly compare the effect of single-drug therapy to the cell and infer the drug combination effect.

The process of generating the OMU system starts by creating a gene regulatory network (GRN) for each drug-cell line combination for both treated and non-treated groups. The GRN is a bipartite graph consisting of two types of nodes: transcription factors (TF) and genes (S1 Fig). The edge between the TF and gene indicates how strong the TF affects the gene in the GRN. Using the GRN, not only are the differentially expressed genes meaningful but also the non-altered genes as all the information of the gene expression profile is integrated into the GRN. A GRN reflects the complex cellular processes and defines the cellular response to a particular phenotype. The differences in GRN structures can reflect the altered biological processes changed between phenotypes (e.g. drug vs no drug). For example, a GRN analysis between cell lines and their tissues of origin has uncovered regulatory differences [15]. Network analysis-based approaches have also been used to find sex-specific regulatory features and differences in regulatory processes involved in drug metabolism between healthy tissues and disease [16,17]. To generate the GRN for our algorithm, we integrated three types of data using the PANDA algorithm [18] for each cell-drug combination. We integrated gene expression profiles, protein-protein interactions (PPI), and gene regulatory relationship datasets to estimate transcriptional networks that distinguish two cellular states (e.g. with or without a drug). PANDA generates accurate GRNs as described by Glass et al. [18].

Next, we extrapolated the differential networks between GRNs of drug-treated and non-treated cell lines. In our algorithm, we are not looking for the individual gene changes or the individual TF-gene alteration. We are searching for the "module" alteration of the GRN in a cell after being treated by a drug. Some GRN subnetworks have a higher density of connectivity than other parts of the network, and these subnetworks are called “modules”. All the genes in a module work together to carry out cellular functions. In response to an external stimulus, an individual TF/gene may change its expression level or switch its co-regulated genes to maintain the dynamic balance of the network. This alteration of the network can be detected by the changing of modules and reflects the phenotypic changes of the whole cell [19]. In this step, we identified changes in the modules by calculating the “differential modularity” in comparison to the drug-treated GRN with non-treated GRN using the ALPACA (ALtered Partitions Across Community Architectures) algorithm [19]. The ALPACA algorithm compares the whole structure of two networks to discover differential gene modules.

**Fig 2:**
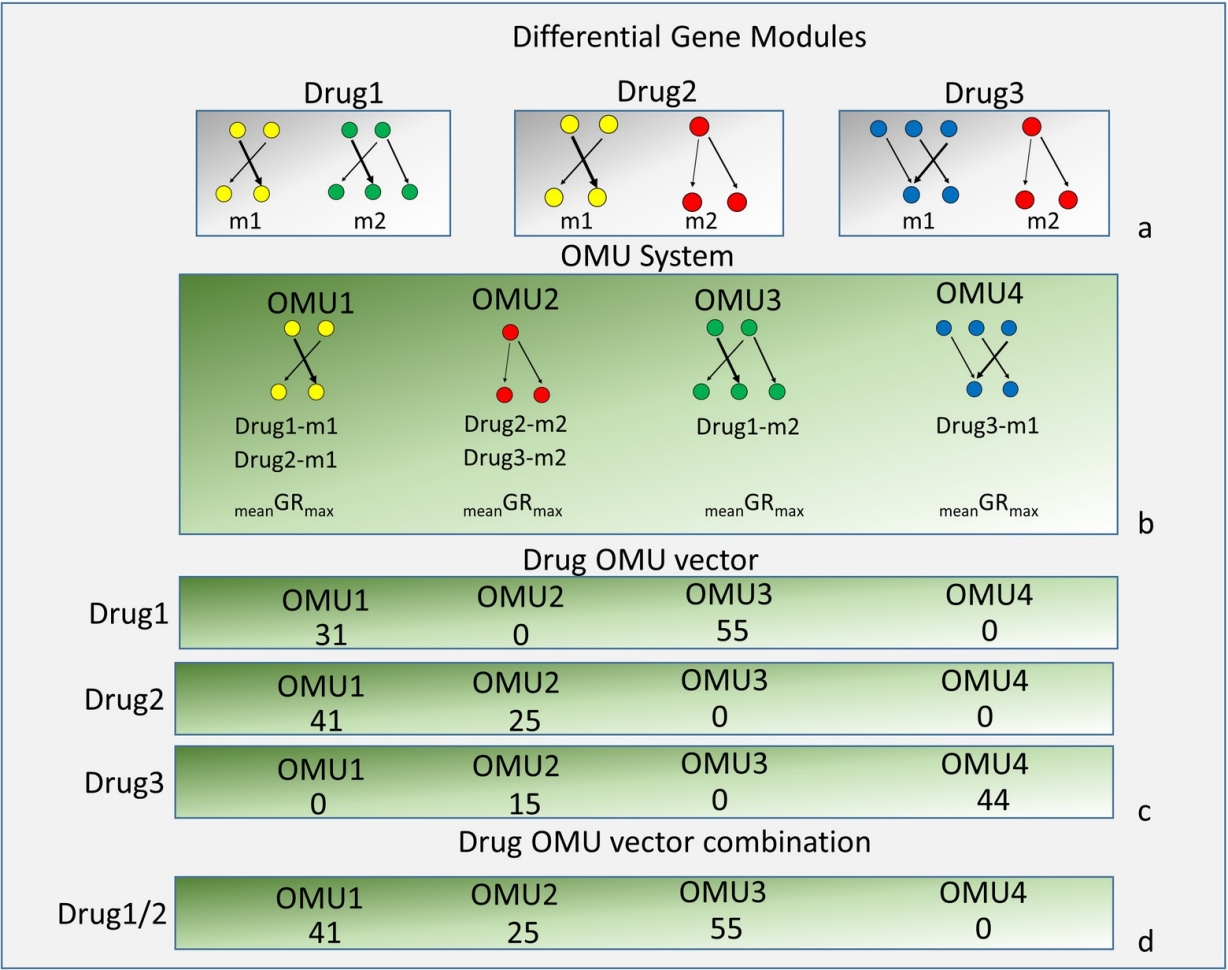
Flow chart to construct the OMU system and drug OMU vectors. (a) Modules of the drug (b) Structure of the OMU system (c) Drug OMU vector (d) Combine two drug OMU vector.

Those differential gene modules were used to construct the OMU system. Each comparison from ALPACA generates a differential gene network that contains many differential gene modules. The similar differential gene modules will correspond to similar phenotypic alterations. We grouped the similar differential gene modules together and refer to them as an OMU. Subsequently, all the differential gene module-based OMUs were used to establish the compendium of OMUs that were identified for the drugs assayed. We call this collection of OMUs an OMU System (Figs 1C and 2B). In order to make the comparisons between different drug combinations possible, each drug-cell differential GRN was converted to an OMU vector. The OMU system is used to quantitatively connect the genomic (GRN) alteration with phenotype changes in the next step (Fig 1D).

We selected a drug-dose response phenotype (growth rate) to use in our algorithm. The growth rate is the most popular phenotype being used to study the effect of drugs on a cell. The growth rate directly reflects the cell’s response to the drug. We used the normalized growth rate inhibition (GR) algorithm which quantifies the cellular response to the drug [20]. The GR algorithm has shown good results for accessing the effects of a drug in dividing cells [20]. GR_max_ measures the maximal drug effect on growth rate and it falls between 1 and -1. The character of the defined range makes GR_max_ suitable for mathematical calculations. To test our algorithm, we selected the breast cancer cell line MCF7 as our model. MCF7 is a well-studied breast cancer cell line and it has been included in many datasets, including LINC. In LINC, MCF7 is one of their five core model cell lines and has been examined by gene expression profiling by L1000 under many perturbations [14]. We randomly selected 57 drugs which have gene expression data in L1000 as our test dataset. All selected drugs were applied to MCF7 respectively to calculate the GR_max_ of the drug. We argue that each differential gene module in a GRN of a cell proportionally represents the GR_max_ of the whole cell. Similar differential modules from various drug treated samples should have similar contributions to the growth rate of the cells. To assign the growth rate value to an OMU, we created a new quantity called “mean GR_max_”, which is the sum of the proportional GR_max_ values of the modules included in an OMU in the OMU system. (See Materials and Methods). In the OMU system, each OMU has three elements: 1) the structure: which presents the network of differential gene modules, 2) the members: indicates which differential gene modules belonged to this OMU, and 3) the mean GR_max_ of the OMU. Based on the mean GR_max_ and using hierarchical clustering, all OMUs were grouped according to their difference in mean GR_max_ and would be used in the next steps to investigate how these OMUs change in each drug treated sample with respect to GR_max_ (Figs 1C and 2B).

In order to explore the relationship between the alteration of the GRN (represent by the OMU vector) and cell phenotype (GRmax) changing, we introduce the concept of balance trees and the isometric log ratio (ILR) transformation. In the OMU vector, the activity levels of each individual OMU are not independent from each other and the change in the relative activity of one OMU could influence the apparent activity of the others. This compositional character of the data makes it hard to find correlations between a single OMU and GR_max_. Additionally, the OMU vectors are sparse vectors (Fig 2D), meaning there are few OMUs presented in all vectors which make it problematic for statistically detecting relationships between an OMU and GR_max_. To solve the compositionality problem, J.J Egozcue *et al* introduced the balance concept as an exploratory technique in geology [21]. Further, Morton *et al* used it in their research of the soil microbiome. They applied the balance and balance tree concept to study the relationship between soil microbiomes and soil pH, which infers the relative changes of microbial sub-communities along with the pH change [22]. We adapted their concept and extended it to the OMU system. Based on the hierarchical clustering of the OMU system, we were looking for the balance (ILR) between the OMUs of the left branch (associated with cell death) and the OMUs of the right branch (associated with partial inhibition) at various points (triangles) (S2 Fig). Balance is the concept we use to show the dynamic changing of the OMU vector. ILR transformation is the mathematical method for calculating the value of the balance. ILR transformations work on compositional data and can transform high dimensional data to 1 dimension, allowing us to apply a linear regression. Based on which point is used to calculate the ILR, different subgroups of the OMUs would be included in the calculation. For instance, the balance using the black triangle only assesses the blue and orange subgroups while the balance calculated using the red triangle includes all subgroups (S2 Fig). Since our OMU vectors are sparse vectors and we wanted to include all the OMUs in our analysis, we used the root point in order to include all the OMUs in our study. The point at the red triangle had been used to obtain the balance between the high mean GR_max_ and the low mean GR_max_ modules. The balance from all drug treated samples was calculated using an ILR transformation and then used to fit a linear regression model with GR_max_. This transformation and linear regression allowed us to not rely on the correlation between differentially expressed genes and GR_max_, but instead inferring the correlation between GR_max_ and the change in OMUs (a change in the balance). This helps us capture the dynamic changes of all OMUs under the branch. The balance between the high mean GR_max_ and the low mean GR_max_ modules are calculated using the ILR transformation.

To find the linear relationship between GR_max_ and the changing of the OMUs in each OMU vector, we first calculated the ILR for each drug OMU vector. Then we used the GR_max_−phenotype of the cell and ILR–genotype of the drug to fit a linear model (Fig 1E). This linear model illustrated the relationship between ILR and GR_max_ which came from a single drug treatment data and would be used to predict two-drug combination effects via computer simulation.

Finally, based on the OMU system and linear model, we created a scoring system to prioritize the two-drug synergistic combinations (Fig 1F–1H). First, we used all the single-drug OMU vectors to create the simulated two-drug OMU vector for all pairwise combinations (Figs 1F and 2D). Second, we calculated the ILR for each simulated two-drug OMU vector. Third, we assessed the “simulated GR_max_” based on the previously derived linear equation. We called this “simulated GR_max_” the Growth Impact Score (GIS) to prevent confusion. We now have the GIS for all pairwise two-drug combinations (e.g. Epirubicin/Vemurafenib) as well as each combination from the single drug paired against itself (e.g. Epirubicin/Epirubicin, Vemurafenib/Vemurafenib). Since our goal was to investigate synergistic drug combinations, we expect the effect of two different drugs to be greater than the sum of their individual effects. We want to find the drug pairs which decrease the GIS. By comparing the GIS from two distinct drug combinations with the GIS from their corresponding single drug combinations, we could estimate their predicted growth change. We call this the differential growth impact score (DGIS) (Materials and Methods). The DGIS is inferred from the GR_max_ with the more negative the value, the higher effect of the drug on growth (cell death). We ranked the two-drug combinations based on their DGIS score with lower scores having a higher ranking. To evaluate how well the algorithm enriched for synergistic drug combinations, we picked the top 30 ranked drug combinations in the DGIS list and used the zero interaction potency (ZIP) model to identify true synergistic combination [23]. The ZIP model is a gold standard and captures the change in the dose-response curves between individual drugs and their combinations to give an estimate of synergy or antagonistic effect. In order to assess our algorithm relative to random chance, we randomly selected 10% (160) of all possible pairwise drug combinations (1596) from the data and tested these combinations using the same ZIP model. These randomly selected drug pairs were used to calculate an enrichment score corresponding to the list of top synergistic drug combinations.

## Materials and methods

### Cell line and tissue culture

Frozen MCF7 cells were obtained from the ATCC and grown according to ATCC recommendations. The cell lines were maintained in DMEM media (Gibco) with 10% serum (Gibco) at 37°C with 5% CO2 in a humidified incubator. Before the experiment, the cell line was passed twice after thawing.

### Measure drug response in MCF7

MCF7 cells were seeded in 96 well plates at 3000 cells per well and grown overnight allowing them to adhere and recovered from trypsin treatment. Cells were treated with a dilution series of the indicated drugs. After 72 hours of incubation, cell viability was measured using the CCK8 assay [20]. Briefly, after removing the medium from the 96 well plate, fresh medium with 5% CCK8 was added to the wells and incubated for 4 hours. Absorbance at 450 nm was measured using a BioTek Synergy H1 microplate reader. The drug growth rate inhibition was calculated following the GR metrics algorithms [20]. The formula to assess growth rate metrics (1) calculates the ratio between growth rates under treated and untreated conditions normalized to a single cell division.


$$GR(c,t)=2^{\frac{k(c,t)}{k(0)}}-1$$ (1)

Where GR(c, t) is the GR value at time t in the presence of the drug at concentration c and k(c, t) is the growth rate of drug-treated cells and k(0) is the growth rate of untreated control cells.

### Synergy scoring and detection

In this study we used a total of 57 cancer drugs to assess the algorithm. A list of the tested drugs with their concentration ranges is shown in S1 Table. The drug-specific concentration ranges were selected based on the single-drug cell response curve in MCF 7 cell line, which were used for defining a range of active and inactive concentrations for these drugs. The drug combinations were tested in a 6 X 6 matrix in MCF 7 cells as described in Bhagwan’s paper [23]. The dose-response matrix data was used to calculate the synergy score, which were calculated using SynergyFinder R package [23,24]. SynergyFinder fit the data in a zero-interaction potency (ZIP) model allowing us to calculate a delta score to assess the drug combinations synergy effect. The summary delta score (ZIP score) is between -1 (antagonist) and 1 (synergy), indicating the percentage of the extra effect of a drug combination compared to the expected effect.

### Network and Operational Module Unit (OMU) system

#### Regulatory gene network reconstruction

We used the PANDA algorithms to construct gene regulatory networks for each drug treated and non-treated cell [18]. The analysis was accomplished in R using the panda function in the pandaR package. The parameters of panda were set as their default value except “mode” was set to “intersection”. The networks were generated from integrating three types of data: 1) TF/target gene regulatory database; 2) protein-protein interaction data; and 3) gene expression data. The TF/target gene regulatory database was combined from three literature curated data bases: Transcriptional regulatory relationships unraveled by sentence-based text-mining (TRRUST) [25], the Open Regulatory Annotation (ORegAnno) database [26], and the Human Transcriptional Regulation Interactions database (HTRIdb) [27]. Protein-protein interaction data was from StringDb v10. To minimize the effect of the false-positive data generated, we only keep the interactions derived from experimental data. The gene expression profiles of the drugs were downloaded from the public LINCS L1000 dataset website (lincscloud.org) [14]. After running the panda function, we extracted the gene regulatory network from the “panda” object for the next step.

#### Differential network generation and module discovery

To assess the altered gene regulatory networks between the drug treated and non-treated cell, the ALPACA (Altered Partitions Across Community Architectures) algorithm was used [19]. ALPACA compares the full network structures active in each condition-specific network to find differential gene modules. ALPACA is comprised of three steps: 1) Finding the modules in the baseline condition using methods based on maximizing the modularity. 2) Calculating the “differential modularity” score. This score compares the number of edges in a module from the drug treated cell to the expected number of edges based on the pre-computed community structure of the base line network from step 1. 3) Apply the Louvain optimization algorithm to iteratively aggregate the nodes into modules [28]. Here, we compared each drug treated cell with the non-treated one, respectively. In each comparison, there were 15 to 19 differential gene modules found. These differential gene modules contained dense communities of genes that carryout cellular functions corresponding to the effects of the drug on the whole gene regulatory network. The edge weight in the module is the differential modularity score, which presents the contribution of the edge to the alteration of the module, and will be used in the next step.

#### Construct the Operational Module Unit (OMU) system and drug OMU vector

Module-based analyses aim to incorporate information about gene connectivity. Under the assumption that genes within modules are cross-correlated, the same gene network should perform similar cellular functions and will contribute to the same cellular phenotype. We applied the Jaccard Index to detect similar edges between the modules. A similar edge is defined as a link in the module with the same nodes on both ends. In Eq 2, J is the Jaccard index which lies between 0 and 1 where 0 represents complete difference and 1 represents the exact same.


$$J(A,B)=\frac{\left|A\cap B\right|}{\left|A\cup B\right|}=\frac{\left|A\cap B\right|}{\left|A|+|B|-|A\cap B\right|}$$ (2)

A and B are two modules, which are generated from the comparisons between drug treated GRN and non-treated GRN. Union of A and B, which is a group of shared edges between modules A and B, is a subnetwork in both modules. We defined these similar gene subnetworks as an operational module unit (OMU). Each module can have multiple OMUs, which reflect the different cellular functions of these modules. At the same time, each differential network contains multiple modules and hence contains multiple OMUs (Fig 2A). All OMUs generated from the data form an OMU system (Fig 2B). In each OMU, they contain three pieces of information: 1) Structure: this represents the network that contains all similar edges of differential gene modules, 2) Member: this stores all differential gene modules containing the edges in the structure parts of this OMU, from various samples, and 3) Mean GR_max_ of this OMU.

Once the OMU system has been built, the OMU vectors for each drug treated sample will be generated according to the location of its modules in the OMU system. (Fig 2C) The drug OMU vector is a sparse vector: an OMU which contains the differential gene modules in the differential network has a non-zero value, which is the sum of differential modularity scores of all edges in the module; the OMU which does not contain a differential gene module has been assigned a zero value.

#### Calculating the mean GR_max_ for each OMU

The mean GR_max_ of each OMU used in this study was calculated as follows: (Eq 3)

$$mean\;GRmax\;(OMU)=\sum_{i=1}^{n}GRi\frac{Xi}{\sum_{j=1}^{m}Yj}$$ (3)

There are n numbers of modules in the OMU. Module Xi is a member in the OMU. GR_i_ is the GR_max_ of the sample i which contains module Xi. In this sample, there are m numbers of modules. X_i_ and Y_i_ is the sum of differential modularity score of all edges in modules i and j.

#### Isometric log ratio (ILR) transformation and linear regression

The core data analysis is to transform compositional data from multiple dimensions into one dimension where standard statistical tools can be applied [21]. The ILR transformation is a method which transforms compositional data into one dimension. We calculated the ILR using the OMU hierarchical clustering based on mean GR_max_ using the following formula:

$$bi=\sqrt{\frac{\left|iL||iR\right|}{\left|iL|+|iR\right|}}log[\frac{g(iL)}{g(iR)}]$$ (4)

where bi is the balance at internal point i, iL is the set of all OMU proportions contained in the left subtree at internal point i, iR is the set of all OMU proportions contained in the right subtree, g(x) is the geometric mean of all the proportions contained in this vector, |iR| is the number of OMUs contained in iR, and |iL| is the number of OMUs contained in iL. For each drug OMU vector, we calculated their ILR at the selected point of the OMU vector. The correlation between ILR and GR_max_ from all samples was calculated. The linear regression between ILR and GR_max_ was performed with lm function in R and a fitted linear model was used to predict the effect of the two-drug combinations.

### Two drug combinations and Differential Growth Impact score (DGIS)

The task of estimating the effect of the drug combinations was implemented using the linear function calculated from the previous step. To accomplish this, we performed the following steps: 1) Single drug OMU vectors were combined pairwise to generate two-drug combination vectors through vector addition in which common OMU elements would take the larger value of the two OMUs from each vector (Fig 2). The new two-drug combination OMU vector had the same length as each single OMU vector. The value of each OMU in the vector was normalized to percentage values of each OMU over the total OMUs in the vector. In addition to creating the two-drug OMU vector, we also calculated a vector combination for each of the single drug OMU vectors with itself (Fig 1F), which was mathematically the same as the single drug OMU vector; 2) We calculated the simulated ILR for all two-drug OMU vectors; 3) By using the linear model from the single drug data, we calculated the Growth Impact Score (GIS) for each two-drug combination OMU vectors based on the simulated ILR; and 4) The differential growth impact score (DGIS) was calculated using the GIS from the two drug combinations compared with the GIS of the two individual single drug vectors (Fig 1F–1I).

**Fig 3:**
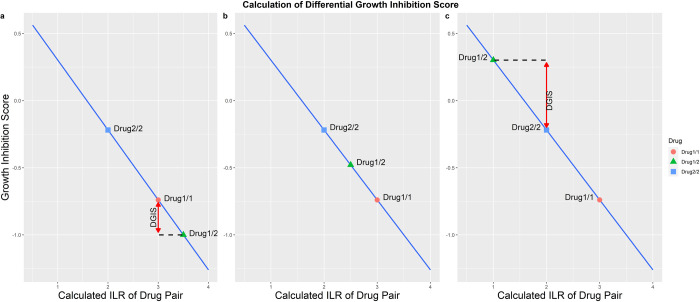
Schematic diagram of how to calculate the DGIS.

An example of how we calculated the DGIS for the two-drug combinations is shown in Fig 3. The calculated two-drug combination DGIS is based on the regression line calculated previously. In this example, we compare where the calculated GIS from the two-drug combination (Drug1/2) is on the regression line relative to the individual single-drug combinations from the same drugs (Drug1/1 and Drug2/2). In this example, single Drug1 has a more negative impact on cell growth than single Drug2. Based on where the calculated GIS for the two-drug combination (Drug 1/2) is relative to the single drugs we can estimate the two-drug combination’s effect on growth. There are three possible locations for the GIS of the two-drug combination (Drug1/2): 1) it can be less than Drug1/1, which indicates the combination of Drug 1/2 tends to have more negative effects on cell growth (i.e. Drug 1 had a more negative effect on growth than Drug 2). The difference of the GIS between Drug 1/2 and Drug1/1 is called the differential growth impact score (DGIS) and will be negative; 2) if the Drug 1/2 location is between Drug1/1 and Drug2/2 on the regression line, this indicates that Drug1/1 and Drug2/2 affect the cell growth independently, the DGIS is set to zero; 3) if the Drug 1/2 location is more than Drug2/2, this suggests that the Drug1/2 combination will have a less negative effect on cell growth and the DGIS will be positive. The DGIS of all pairwise drug combinations was calculated based on the effect of the two-drug combinations on growth rate relative to the individual single drugs.

## Results

### The GR_max_ of anticancer drugs show heterogeneity in their cellular response

**Fig 4:**
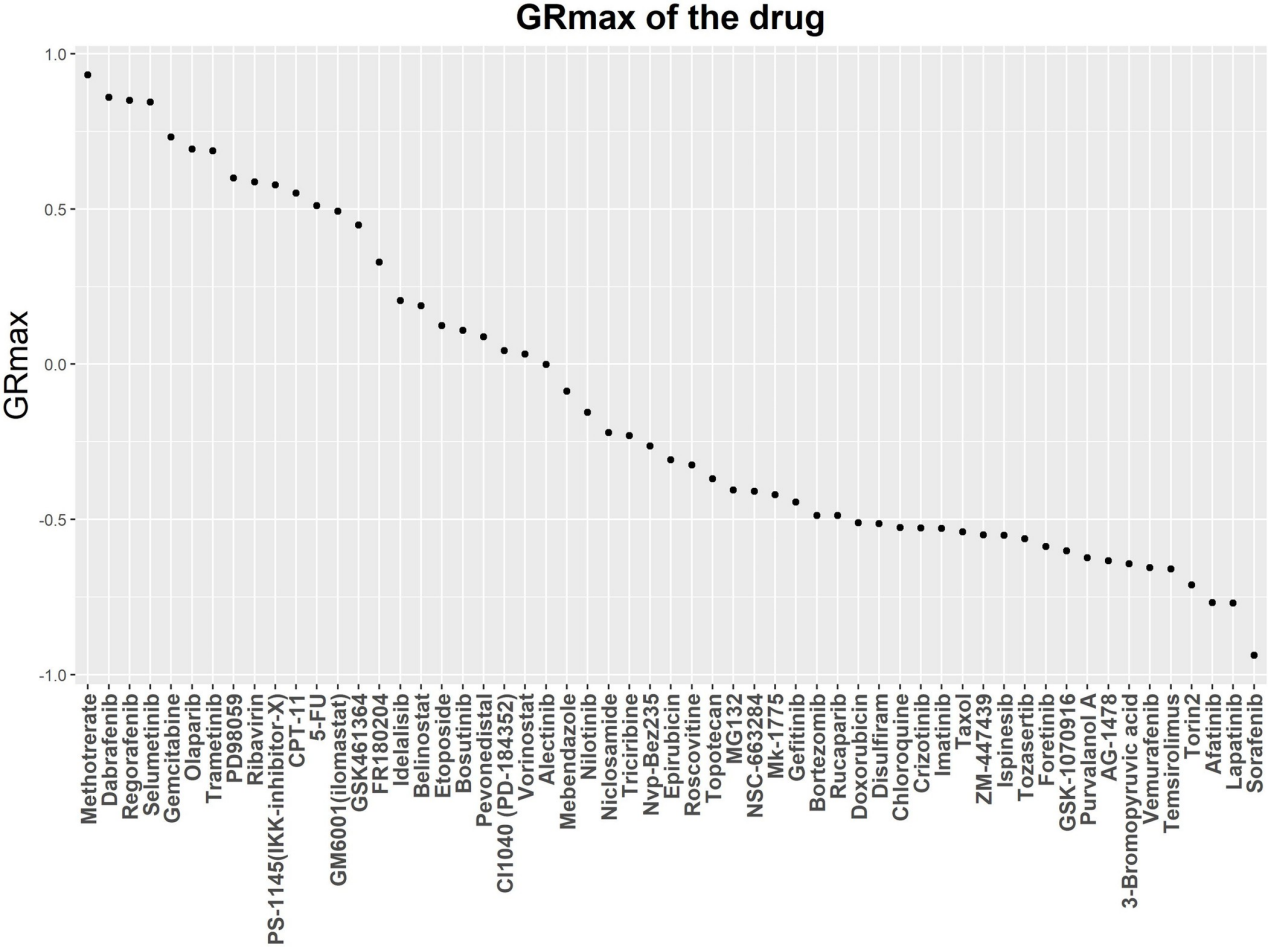
GRmax distribution of the 57 drugs sorted by GRmax value.

We selected the GR_max_ from the growth rate (GR) inhibition algorithm as an indication of the cellular response to anticancer drugs (20). GR_max_ captures the maximal drug effect on growth rate and has a defined range of -1 to 1. It can easily be integrated into our algorithm in order to calculate the mean GR_max_ of the OMUs and has an interpretable meaning on cell growth. A total of 57 randomly selected anticancer drugs were applied to the breast cancer cell line MCF7. Fig 4 presents the GR_max_ of each drug used in the study. The distribution of the calculated GR_max_ on MCF7 cells indicates that most drugs either inhibit cellular growth (0 < GR_max_ <1) or kill the cells (-1< GR_max_ < 0). A number of drugs had GR_max_ close to 1, suggesting they do not substantially affect cellular growth of MCF7. Including these drugs in the analysis could potentially expand the number of possible candidate drug pairs. Our data showed no bias in the range of GR_max_.

#### Reconstruct single drug treated cell gene regulatory networks and generate differential networks

We performed a gene regulatory network (GRN) analysis using PANDA in order to construct gene regulatory networks for each individual drug-cell combination and determined the differential networks between the single drug-cell combination and their non-treated counterparts using ALPACA [18,29]. In the cell, genes interact with each other to form a network in order to maintain normal cellular functions. Cellular gene networks would be altered in response to external environmental changes (i.e. a drug) in order to maintain cellular continuity. These alternate networks include not only the differential gene expression changes but also the gene regulatory changes, which contained translational factors switching their partners instead of changing their expression level as well as the translational factors changing their targeted genes. Gene regulatory network analysis can detect these alterations and discover the relationship between biological phenotype and genotype.

PANDA uses a message-passing algorithm that utilizes a non-weighted TF-GRN and integrates protein-protein interaction data and gene expression data to form a weighted TF-GRN. In the weighted TF-GRN, each edge connects a TF to a target gene, and the edge weight reflects the strength of the regulatory relationship (S1 Fig). In our data, each GRN contained 833 genes and TFs and total 167620 edges. We determined 57 GRNs from drug treated cells and 1 GRN from non-treated cell. The weight of the edges was a z-score calculated by setting parameter “zScale = TRUE” when we ran PANDA in R.

We then applied ALPACA to compare each of the drug treated GRNs with the non-treated GRN. ALPACA compares the full network structures active in each condition, computes a differential modularity matrix D_ij_ for the treated network relative to the non-treated network and determines differential gene modules through a Louvain algorithm [19]. A total of 57 comparisons were done and each comparison generated 15 to 18 differential gene modules with a total of 866 modules determined for all the drug-cell combination data. Each differential gene module comprised various nodes (genes) and edges that contributed to the altered cellular response of the MCF7 cell under each drug treatment.

### Creating the Operational Module Unit (OMU) system, OMU vector, and calculating the mean GR_max_

**Fig 5:**
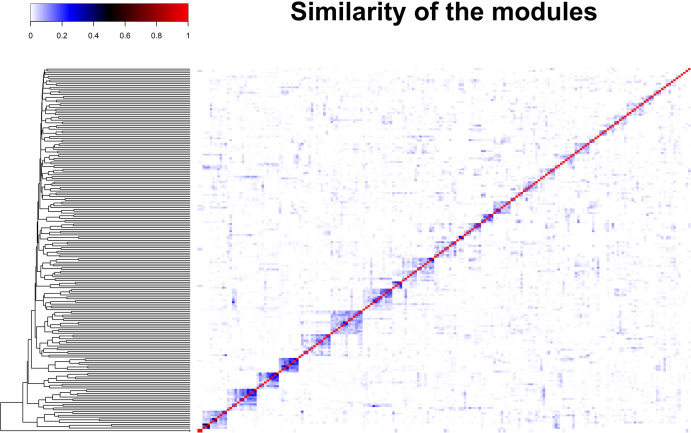
Heat map showing the similarity of the differential gene modules. Both x and y axis are individual modules.

**Fig 6:**
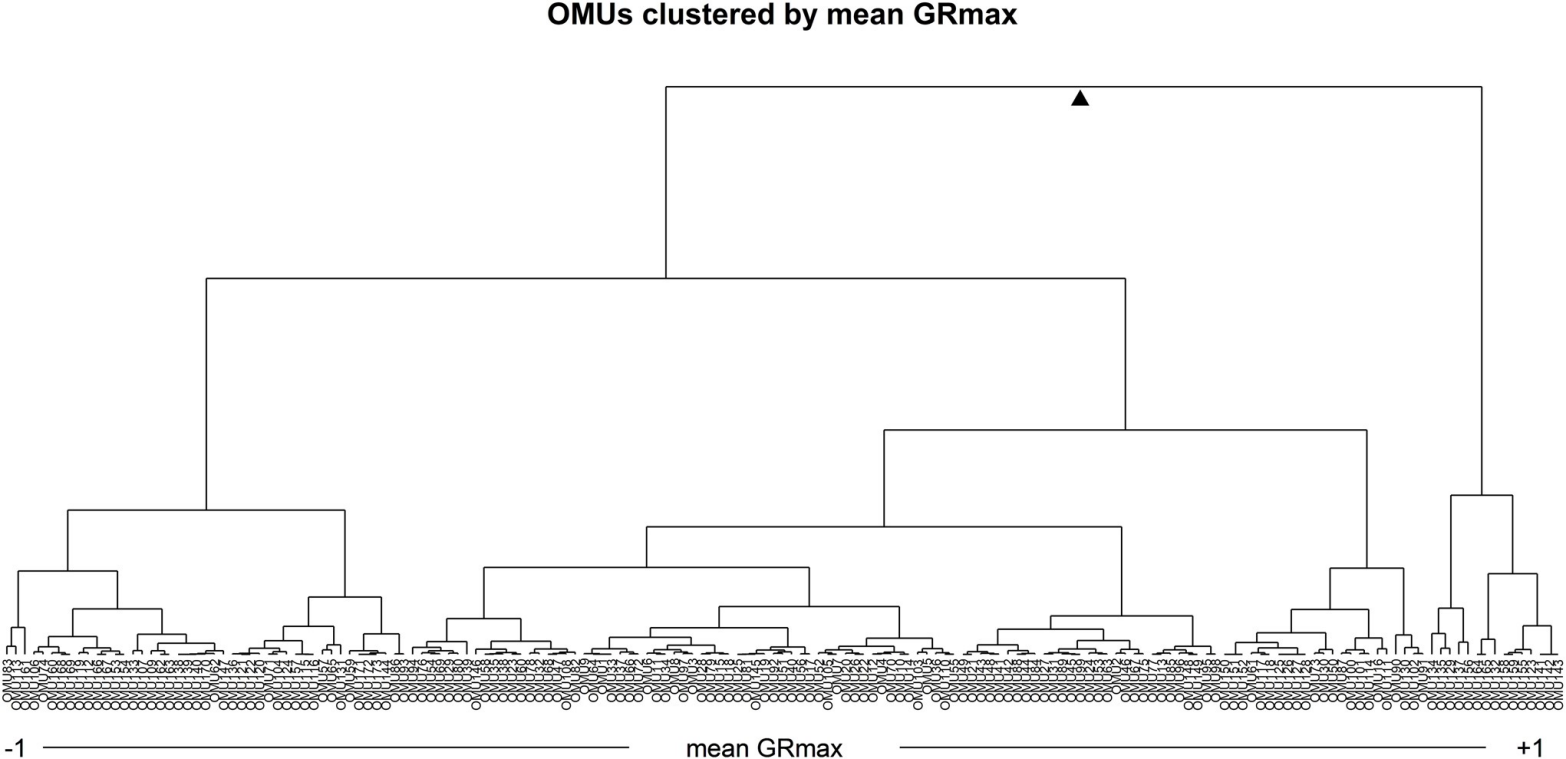
Hierarchical clustering of the OMUs in the OMU system based on mean GR_max_.

We created the OMU system based on the similarity of the differential gene modules (Fig 5). Similar edges are the edges in the differential gene network which link the same TF and gene. The OMU is defined as a sub network which includes a group of similar edges. We started with the similarity matrix of the differential gene module and sorted the module pairs based on the similarity score from highest to lowest. In this way, we could increase the specificity of the OMU. The pseudocode of construction of the OMU system is shown in S2 Table. After building the OMU system, the mean GR_max_ of each OMU was calculated based on Eq 3. We performed hierarchical clustering of all OMUs based on their mean GR_max_ in order to understand the OMUs relationship to their mean GR_max_ value (Fig 6).

**Fig 7:**
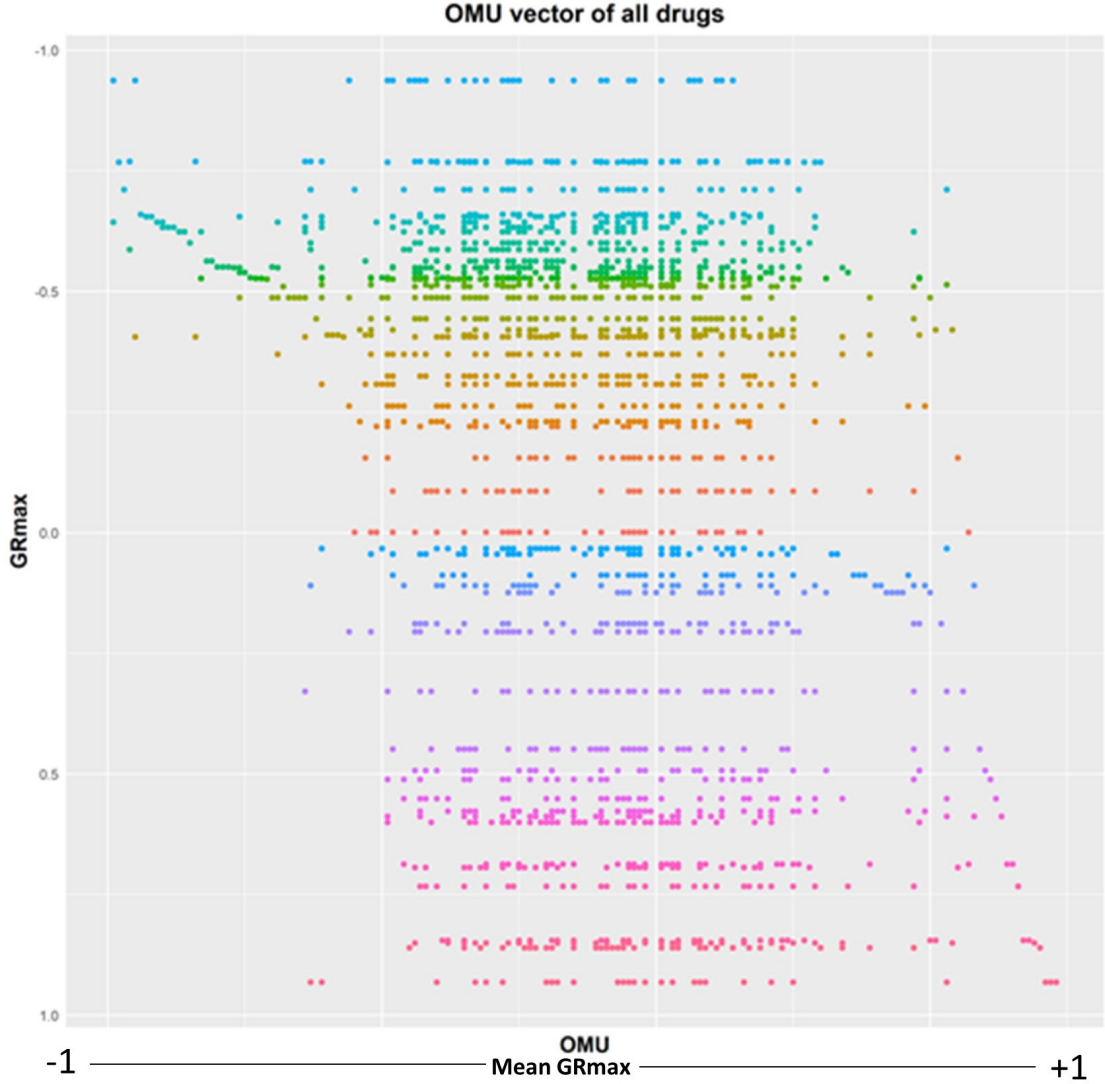
The OMU vectors for all 57 drugs.

Once the OMU system was built, we next generated single drug-cell OMU vectors. Briefly, each differential gene network had multiple modules and these modules belong to different OMUs in the OMU system (Fig 2). The location of the modules in the OMU system indicated which OMUs had value. Finally, all 57 drug-cell differential networks were transferred to 57 OMU vectors. Since all OMU vectors contained zero values, they were all sparse vectors. All OMU vectors were plotted together based on mean GR_max_ of the OMU and GR_max_ of the drug (Fig 7). We noticed that some OMUs were common and found in most OMU vectors while several OMUs were unique and appeared only in one combination. A clear trend could also be observed in Fig 7, namely that the OMU vectors of the drugs with negative GR_max_ (negative growth) tended to have more OMUs correlated with negative mean GR_max_. To further investigate this tendency and relationship between OMU and GR_max_, we performed an ILR transformation of the OMU vector.

### Isometric log ratio (ILR) transformation and linear model

The weighted regulatory gene network built by PANDA used data from three different databases, including gene expression data. The gene expression data quantifies the relative abundance of individual gene activity and reflects how genes change in response to a drug. The compositional nature of the data inherent in the OMU system provides a challenge for directly applying statistical methods on them. As mentioned before, ILR transformation of the OMU system can transform high dimensional data to a one dimensional form and be analyzed by standard statistical methods [19].

**Fig 8:**
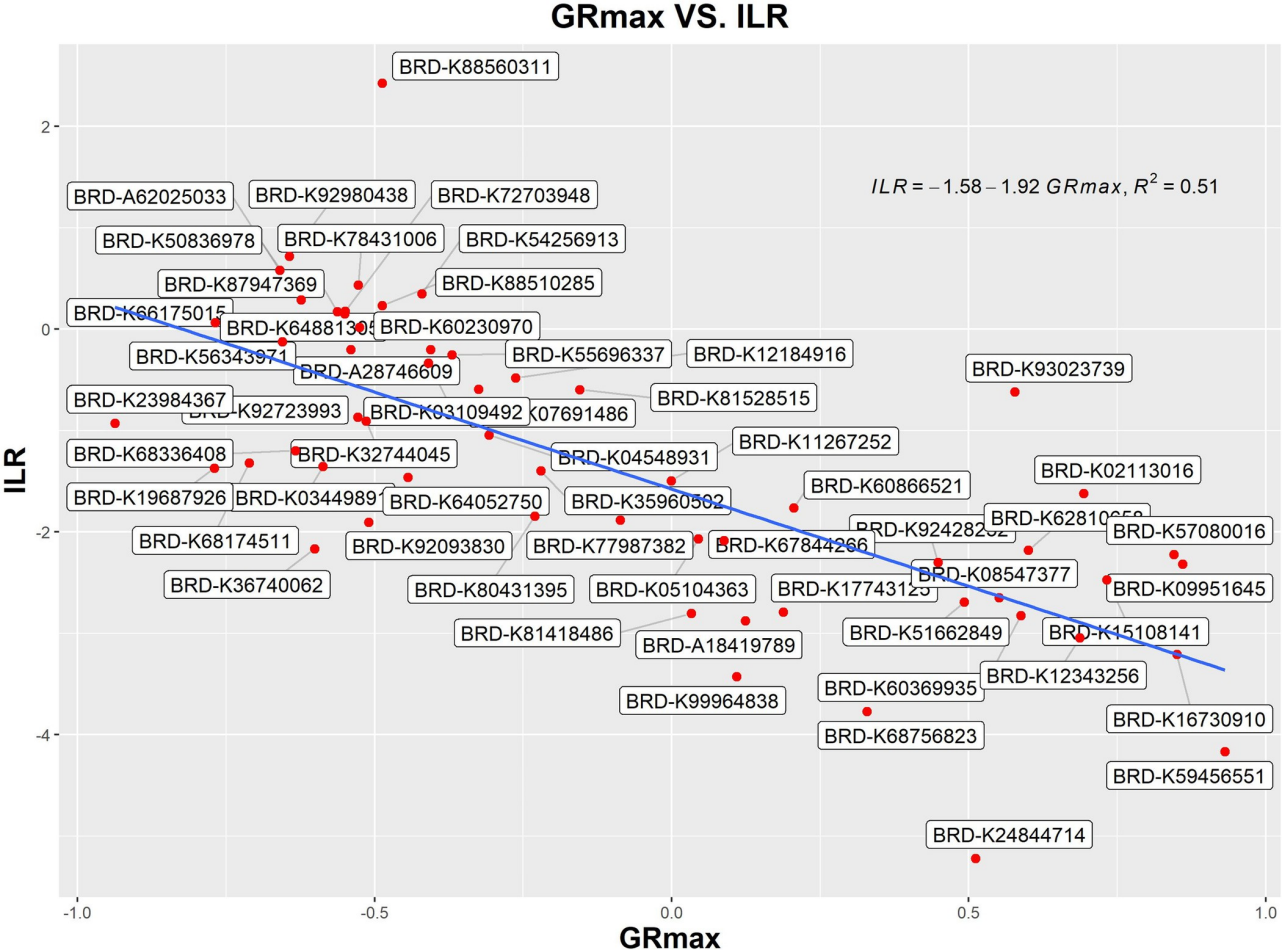
Linear regression of ILR of a drug OMU vector against GRmax of the same drug.

In order to accomplish the ILR transformation, we need to pick a balance point on the mean GR_max_ hierarchical cluster of the OMU vectors that allows us to assess the modules that inhibit growth relative to the modules that don’t. From Fig 6, we determined that some OMU vectors in the range of mean GR_max_ less than -0.5 or greater than 0.5 do not have non-zero OMUs. Since ILR transformation requires non-zero values on both sides of the balance point, we picked all points which had mean GR_max_ between -0.5 and +0.5 to calculate the ILR for all drugs and determine the optimum balance point for this OMU cluster. At each point, the ILR was calculated for all 57 drug OMU vectors. To identify the optimal balance point, each set of ILRs generated at each cut point was used to calculate the correlation between ILR and GR_max_ of the drug. The optimal balance point was determined to be at OMU92, which had mean GR_max_, of -0.140 and is negatively correlated with the overall GR_max_ (r = -0.714). (Fig 8). Finally, this optimal balance point was used to calculate the ILR of each drug-cell combination and fit a linear model (Fig 7). Through ordinary least-squares analysis on the ILR, the GR_max_ could be predicted via ILR alone with an R^2^ of 0.51, which suggested that ILR alone explained over 51% of the total variation in the entire differential network. This linear model was used to predict the effect of the two-drug combinations.

### Generate the differential growth inhibition score (DGIS)

**Fig 9:**
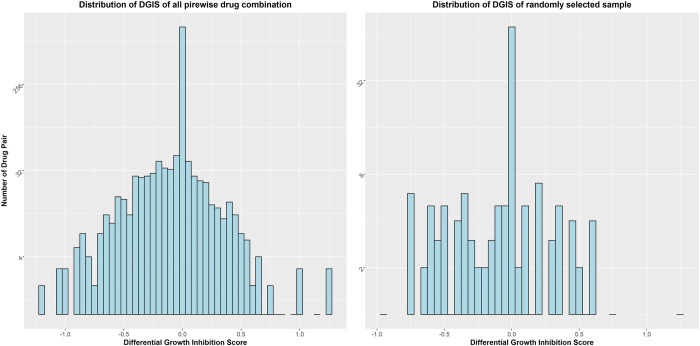
Distribution of DGIS for all pair wise drug combinations.

From the previous section, we determined the linear model that reflects the relationship between the gene network (ILR) and phenotype (GR_max_) from the single drug treated cells. Next, we used this model to calculate the DGIS for the two-drug combinations using the following steps. 1) We calculated all pairwise single drug OMU vectors (two drug combinations) and updated the values for each OMU element in the vector by using the max value between the two single drug OMUs in the combined OMU vector (Fig 2). We assumed that each drug individually affected the OMU when combining the two drugs. If an OMU was found in only one drug, the combined value would be the value from this drug. If an OMU was found in both drugs, we used the value with the larger affect. All 1596 two-drug pairwise combined OMU vectors were generated as well as 57 OMU vectors for the samples treated with a single drug for a total of 1652 OMU vectors that represent all possible combinations. 2) The ILR for all two-drug pairwise combinations was calculated using Eq 4 at the optimal balance point on the OMU cluster determined previously. 3) Based on the linear equation (Fig 8) and ILR, we calculated the GIS of all the pairwise drug combinations. Since we combined two drug-cell OMU vectors, the predicted GR_max_ value was no longer limited to -1 to +1. Therefore, this calculated value was designated as the growth inhibition score (GIS), which indicates how much impact the two drugs would have on MCF7 growth when treated together. 4) Our goal is to find the synergistic drug combinations in the drug pairs. A synergistic effect means the inhibited growth rate of the combined drugs is greater than what is expected from the single drugs. We compared the GIS of the two-drug pair combinations with their corresponding single drug pairs (Fig 3) and calculated the DGIS for each two drug pair combination. The distribution of the DGIS from 1596 drug pair showed an approximately normal distribution (Fig 9). It was not surprising that most of the DGIS fell in the zero bin which indicated that most of the drug combinations did not have any synergistic effect. A negative DGIS suggests the two drug combinations will tend to have a greater effect on cell growth compared with the single drugs alone. To verify the true synergistic drug combination, we selected the top 30 drug combinations (2% of the total combinations) with the most negative DGIS scores to check if they had a synergistic effect on cell death using the ZIP model to calculate the synergistic score (Table 1).

**Table 1:** The top 30 drug combination candidates.

drug_1	GRmax_1	drug_2	GRmax_2	DGIS	ZIP
Irinotecan	0.55	Selumetinib	0.85	-1.19	-4.355
PD-184352	0.04	Sorafenib	-0.94	-1.18	-2.113
Bosutinib	0.11	Niclosamide	-0.22	-1.05	1.531
Vemurafenib	-0.66	Vorinostat	0.03	-1.04	-0.932
Epirubicin	-0.31	Vemurafenib	-0.66	-0.99	9.855
Bortezomib	-0.49	Foretinib	-0.59	-0.98	-0.807
Alectinib	0	Irinotecan	0.55	-0.91	160
Foretinib	-0.59	Vorinostat	0.03	-0.9	2.107
Bortezomib	-0.49	Epirubicin	-0.31	-0.89	-2.129
Bortezomib	-0.49	Vorinostat	0.03	-0.87	3.605
3-Bromopyruvic acid	-0.64	Vemurafenib	-0.66	-0.85	24.096
Purvalanol-A	-0.62	Tozasertib	-0.56	-0.84	1.198
Bortezomib	-0.49	AG-1478	-0.63	-0.81	9.904
Afatinib	-0.77	Crizotinib	-0.53	-0.8	-0.154
Sorafenib	-0.94	Topotecan	-0.37	-0.78	-3.851
Bortezomib	-0.49	Trametinib	0.69	-0.76	-2.344
Epirubicin	-0.31	Sorafenib	-0.94	-0.72	24.209
MG-132	-0.41	Tozasertib	-0.56	-0.7	4.404
Roscovitine	-0.33	Vorinostat	0.03	-0.69	0.066
Afatinib	-0.77	Niclosamide	-0.22	-0.69	0.057
Niclosamide	-0.22	Nvp-Bez235	-0.26	-0.68	1.459
Imatinib	-0.53	PD-184352	0.04	-0.67	0.796
Epirubicin	-0.31	Temsirolimus	-0.66	-0.67	0.572
Epirubicin	-0.31	AG-1478	-0.63	-0.65	6.617
3-Bromopyruvic acid	-0.64	MG-132	-0.41	-0.64	2.356
Etoposide	0.12	Selumetinib	0.85	-0.64	1.07
Paclitaxel	-0.54	Vemurafenib	-0.66	-0.6	-1.369
Epirubicin	-0.31	Vorinostat	0.03	-0.59	10.066
AG-1478	-0.63	Vorinostat	0.03	-0.59	8.697
Afatinib	-0.77	Mk-1775	-0.42	-0.59	0.063

GIS, growth inhibition score; DGIS, differential growth inhibition score; ZIP, ZIP score.

### Zero interaction potency (ZIP) test and data analysis

**Fig 10:**
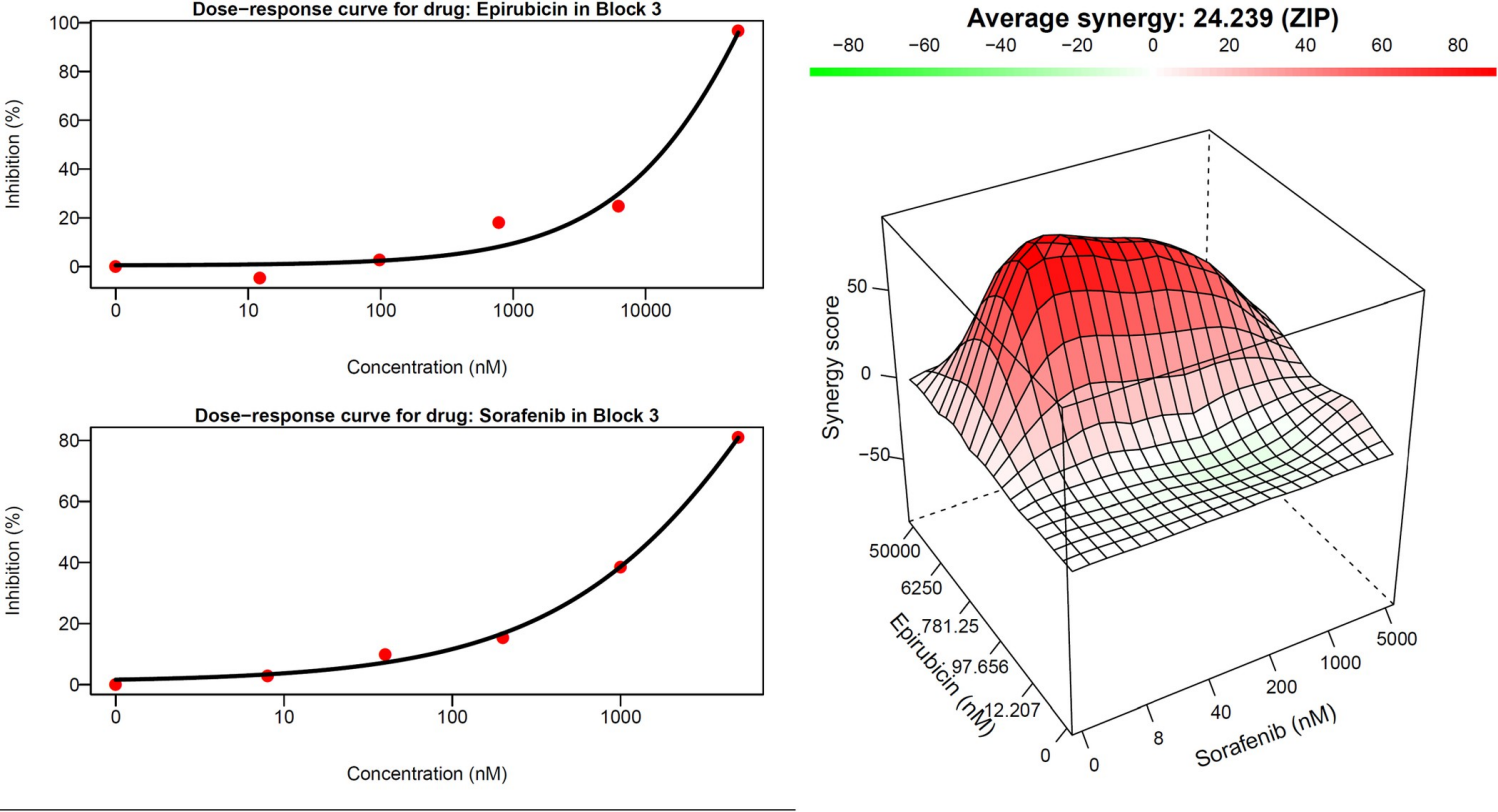
The synergistic drug combinations of Epirubicin and Sorafenib.

To evaluate the top 30 drug pairs, we used the ZIP model to test their combinatorial effect. The ZIP model is the most popular model for testing drug synergy and it is easy to convert the ZIP score to other synergy scores for comparisons [23]. All drug combinations were tested in 6 x 6 dose-response matrix experiments. The raw data was used to fit the ZIP model in order to calculate the synergy score (ZIP score). A positive ZIP score represents a cell response above expectations. For example, when Epirubicin and Sorafenib were combined together, they had a 24% (ZIP score = 24.24) increase above the expected effect suggesting they have a synergistic effect (Fig 10). Using the ZIP model, drug combinations were considered to have a synergistic effect when the ZIP score was greater than 5 [23]. From 30 drug combinations candidates, there were 8 (27%) with a true a synergistic effect (Table 1). Previous experiments and research told us that finding synergistic drug combinations in a randomly selected dataset was rare [6,30]. To determine if our algorithm successfully prioritizes synergistic drug combinations, we compare it with a randomly selected group of pairwise drug combinations from the pool of all possible pairwise combinations.

Since the number of drug combinations in our dataset with true synergistic effect are unknown, we randomly selected 10 percent (160) of all possible pairwise drug combinations (1596) from the list of 57 drugs and determined the ZIP score for all 160 random two-drug combinations. Fig 9B provides the DGIS distribution of this randomly selected cohort. The distribution of this randomly selected subset reflects the distribution of the whole dataset (Fig 9A) and exhibited no bias to any side of the distribution and can be utilized to infer the distribution of the whole dataset. From these select 160 drug combinations, we found two drug combinations that exhibited a synergistic effect (S3 Table). Interestingly, these two drug combinations also appeared in our top 30 list. Since we don’t know the exact number of the real synergistic drug combinations in our drug pair pool, we want to test the null hypothesis that our algorithm will produce the same results as random sampling. The value of M (the true proportion of the synergistic drug combination in the population) that maximizes the joint probability of drawing k = 8 hits in s = 30 samples in one trial and drawing k = 2 hits in s = 160 samples in a second trial is 0.052, with a joint probability of 4.43e-07, under the null hypothesis. That means even though we don’t know the exact value of M, under the null hypothesis, the highest probability of the observed data is 4.43e-7 when M equals 0.052 (83 synergistic drug pairs of out 1596 pairs). Therefore, we determined that using our algorithm to identify synergistic drug combinations is significantly better than randomly picking from the candidate pool and can successfully prioritize synergistic drug combinations.

## Discussion

Cancer is a complex disease where treatment using anticancer drugs often leads to drug resistance. As a result, there is a rising demand for more effective therapies. Using synergistic drug combinations of two or more drugs can overcome toxicity and side effects which can be associated with high doses of single drug therapies. In recent years, several drug combination therapies show promising synergistic effects and have been approval by the FDA [31,32]. Here, we developed a gene network-based algorithm, using both lab data and public data, to successfully prioritize and enrich for synergistic two-drug combinations from a group of candidate drugs and presented opportunities for further exploring multiple drug combinations.

In this study, we have developed an efficient computational-experimental approach to help us study the synergistic drug combinations and use 57 single drug dose-response measurements as a proof of principle. We have illustrated this approach with a randomly selected drug panel treating the MCF7 cancer line. From the top 30 drug combination candidates prioritized by our algorithm (2% of the total possible pairwise combinations), 8 of them were confirmed to have true synergistic effects using the ZIP model. This is significantly better when compared with randomly selecting the drug combinations. Although we cannot claim that this algorithm will find all possible synergistic combinations in a drug panel, it does help us dramatically decrease the cost and time of drug combinatorial screening. Our pipeline showed that combining the individual drug-induced gene expression profiles using GRN structures can infer the effects of the drug pair, which is in agreement with previous studies [8,9]. However, our pipeline differs from previous gene expression based algorithms in that we do not filter the genes. Instead, we use all the genes in our pipeline. Since the differently expressed gene (DEG) profile in a drug combination therapy are distinct from the DEGs observed in a monotherapy, our pipeline has the advantage of using the full set of gene expression profiles integrated into GRNs and contains a large proportion of the information for the drug effect [11]. Notably, researchers can use our algorithm with publicly available molecular profiles of the cancer cell and in house single drug dose-response data to capture the most probable synergistic combinations in their drug panel, and avoid testing all pairwise multiple-doses using a combinatorial matrix hence decreasing cost.

Another advantage of our algorithm is the possibility to increase the number of drug candidates in a drug combination screening. With the aim of less time consuming and cost effectiveness for high-throughput combinatorial screening, several computational approaches have been described. Lanevski et al. [5] developed a computational mode to predict drug combinations using a submatrix of the multiple-dose matrix instead of the full matrix, but this will not solve the problem of the exponential increased number of drug combinations as new drugs are assessed. The submatrix design still requires assessing all pairwise experiments and very extensive experimental work. These extensive requirements of resources usually lead the researchers to include a pre-selecting step to narrow down the drug candidates to a relatively small number. The only input needed for our algorithm is single drug dose-response data to calculate GR_max_, because the protein-protein interaction database, TF-gene database, and molecular profiles of the single drug database are publicly available and updated regularly. That means all information needed in this pipeline is based on the cell and cell response to a drug. Without the need for structural or target information of the compounds, the potential candidates of the combination are not limited to the well-studied compounds. Furthermore, there are a few drugs in our test drug panel that have a flat dose-response curve which implies that these drugs have no effect on the growth of MCF7 cells alone. In traditional screening methods, those compounds usually are filtered out from the list for drug combinations due to no effect of the phenotype at single drug format, but they still can be integrated to our model and directly tested for combinatorial effects with other drugs. While we did not find these synergistic combinations with those drugs in our panel, considering our drug panel is small, there is still a possibility these drugs may have synergistic effects with other drugs. Our algorithm can avoid the pre-selecting step for most traditional high-throughput methods and greatly extends the number of candidates for drug combination screening.

Additionally, our algorithm allows researchers to easily share and reuse the data in the science community for future drug combination searching. Since our algorithm relays only on experimentally derived single drug-cell response matrix, all the data is capable of being stored and reused for running our algorithm again with new data. If a researcher wants to screen their own group of compounds along with the compounds in the database, they can download the data for the previously analyzed compounds and only provide the single drug-cell response experiment and gene expression profile for the new compound in the lab. After that, all they need is to combine the data and run the pipeline. Due to adding any new compound to the drug panel only requires the GR_max_ and molecular profiles from this compound, the additional required resources for extending the screening drug panel increases linearly instead of exponentially. It is much cheaper and faster to screen a large panel of compounds. At the same time, the new compound data generated from different labs can be added to the database and shared with other researchers. Furthermore, once the database extends by including more drugs and cell lines, the future combination screening will become quick and powerful. Each researcher can investigate the combinatorial effect of their interested compounds with all previously studied compounds.

Lastly, our algorithm connected the GRN (genotype) and GR_max_ (phenotype) directly, it is therefore independent from the cell type and the method to measure phenotype. Thus, this algorithm will be robust and compatible with various experimental designs, making it applicable to other biomedical problems, such as antibacterial, antifungal, and antiviral drug combinations synergies. Also, this study solely focused on pairwise combinations on one cell line, but in the future, we will consider higher-order combination prediction and multiple cell lines as well.

## Supporting information

S1 Fig Sample of a GRN. (TIF)

S2 Fig Schematic figure of a balance tree. (TIF)

S1 Table Information of all 57 drugs. (XLSX)

S2 Table Pseudo code of construction of OMU system. (DOCX)

S3 Table ZIP scores of 160 drug pairs. (XLSX)
