# Supplementary figures and images for: A novel network based linear model for prioritization of synergistic drug combinations
Source: PLoS One. 2022 Apr 5;17(4):e0266382. doi: 10.1371/journal.pone.0266382 (PMC8982899; doi:10.1371/journal.pone.0266382)

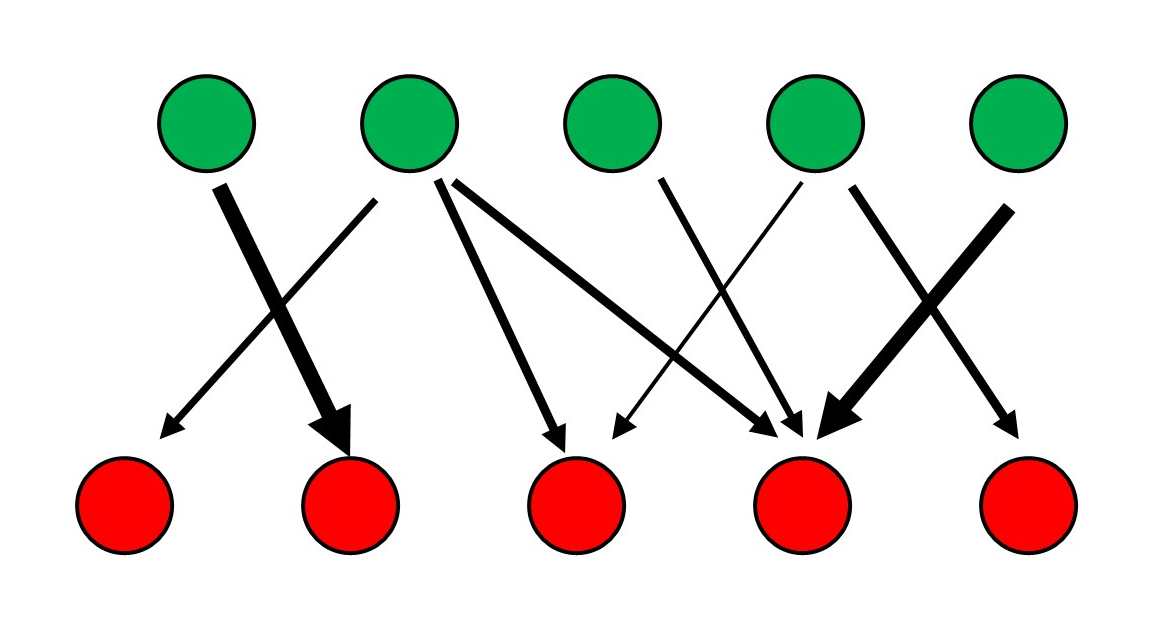

Supplement: S1 Fig — (TIF) [file pone.0266382.s001.tif]

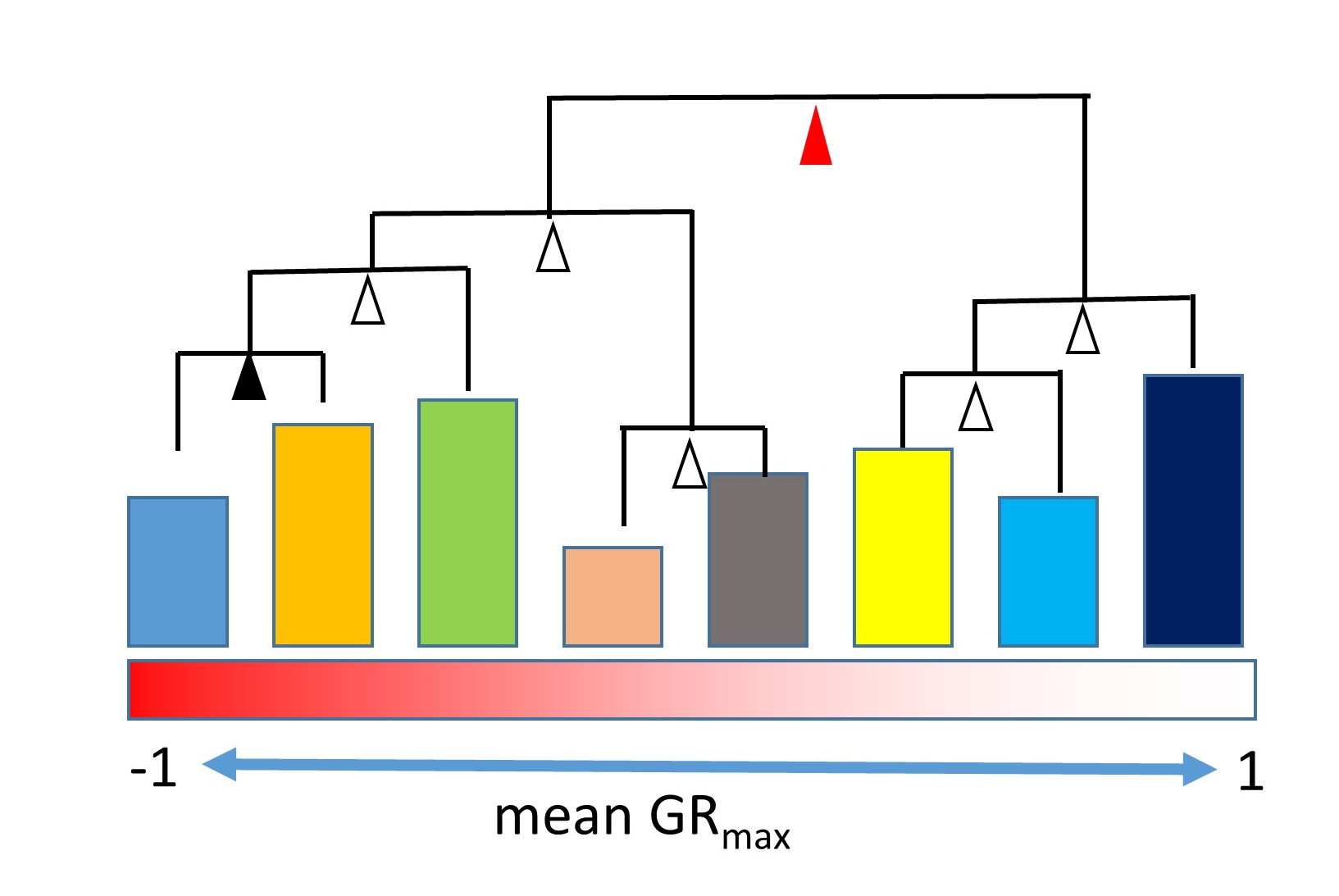

Supplement: S2 Fig — (TIF) [file pone.0266382.s002.tif]
