# Supplementary material for: A novel network based linear model for prioritization of synergistic drug combinations
Source: PLoS One. 2022 Apr 5;17(4):e0266382. doi: 10.1371/journal.pone.0266382 (PMC8982899; doi:10.1371/journal.pone.0266382)
Supplement: S2 Table — (DOCX) [file pone.0266382.s004.docx]

**Construction of an OMU system**

Pseudocode:

Initialize OMU system:

OMU.structure

OMU.member

OMU.meanGRmax

The pairwise modules similarity list:

N pair of modules

Start of

First pair of module from module similarity list:

module 1 (m1) and module 2 (m2)

Compare $m1\cap m2$ with each current OMUs in the OMU system

If $m1\cap m2$ is same as one of exist OMU_x_.structure

Assign m1 and m2 to OMU_x_.member

Else

Create a new OMU_new_

Assign $m1\cap m2$ to OMU_new_.structure

Assign m1 and m2 to OMU_new_.member

Next pair of module

End loop
